# Gender Difference in Renal Blood Flow Response to Angiotensin II Administration after Ischemia/Reperfusion in Rats: The Role of AT2 Receptor

**DOI:** 10.1155/2016/7294942

**Published:** 2016-03-10

**Authors:** Maryam Maleki, Mehdi Nematbakhsh

**Affiliations:** ^1^Water and Electrolytes Research Center, Isfahan University of Medical Sciences, Isfahan 81745, Iran; ^2^Department of Physiology, Isfahan University of Medical Sciences, Isfahan 81745, Iran; ^3^Isfahan MN Institute of Basic and Applied Sciences Research, Isfahan 81546, Iran

## Abstract

*Background*. Renal ischemia/reperfusion (I/R) is one of the major causes of kidney failure, and it may interact with renin angiotensin system while angiotensin II (Ang II) type 2 receptor (AT2R) expression is gender dependent. We examined the role of AT2R blockade on vascular response to Ang II after I/R in rats.* Methods.* Male and female rats were subjected to 30 min renal ischemia followed by reperfusion. Two groups of rats received either vehicle or AT2R antagonist, PD123319. Mean arterial pressure (MAP), and renal blood flow (RBF) responses were assessed during graded Ang II (100, 300, and 1000 ng/kg/min, i.v.) infusion at controlled renal perfusion pressure (RPP).* Results.* Vehicle or antagonist did not alter MAP, RPP, and RBF levels significantly; however, 30 min after reperfusion, RBF decreased insignificantly in female treated with PD123319 (*P* = 0.07). Ang II reduced RBF and increased renal vascular resistance (RVR) in a dose-related fashion (*P*_dose_ < 0.0001), and PD123319 intensified the reduction of RBF response in female (*P*_group_ < 0.005), but not in male rats.* Conclusion.* The impact of the AT2R on vascular responses to Ang II in renal I/R injury appears to be sexually dimorphic. PD123319 infusion promotes these hemodynamic responses in female more than in male rats.

## 1. Introduction

Restricted blood supply to the organ and then restoration of blood flow and reoxygenation are considered as ischemia/reperfusion (I/R). This model reproduces the clinical model of kidney transplantation [1, 2]. I/R may lead to providing reactive oxygen species (ROS), chemokines, cytokines, and leukocytes activation which disturb renal blood flow (RBF) [3, 4]. In addition, I/R leads to endothelial dysfunction which also affects RBF [3, 5]. There is major difference in gene expression between male and female due to physiological dimorphisms [6]. Renal diseases and hypertension are affected by gender in developing and progressing rate [7]. There are also evidences that I/R-induced acute renal failure is gender dependent [8–11], while females are more resistant to I/R-induced kidney injury [12]. There are some factors to be associated with sex difference in I/R-induced injury [13] such as nitric oxide (NO) synthase (NOS) mRNA expression rate [10, 14, 15], endothelin level [16, 17], and sex difference in renal sodium-potassium ATPase [12, 18].

Renin-angiotensin system (RAS) also acts differently between two sexes [19, 20]. RAS is one of the major regulatory systems for blood pressure, fluid, and electrolyte balance. In this system angiotensin converting enzyme (ACE) converts angiotensin I (Ang I) into the active form of Ang II [21, 22]. Ang II acts through two main receptors types 1 and 2 (AT1R and AT2R). Against the effects of AT1R as a strong vasoconstrictor, AT2R was known as a part of vasodepressor arm of RAS [23]. Some studies indicated that the hypotensive effect of AT1R blockade is attributed to AT2R stimulation [24, 25], and the anti-inflammatory role of this receptor is pointed out in renal ischemia [26]. Matavelli et al. reported an early increase in renal AT2R expression after ischemia which revealed the important role of this receptor in the injury-healing process of I/R [27]. It is reported that AT2R blockade attenuates renal production of NO and cyclic guanosine 3,5-monophosphate (cGMP) [28].

Supplying suitable RBF is extremely important to attenuate the renal tissue damage after I/R [29], and, in this regard, Ang II receptors and gender are the main two parameters [30] that need to be considered. In normal condition, the important role of AT2R on RBF response to Ang II was reported by Hilliard and coworkers [20]. Accordingly, we hypothesized that, in I/R condition, the role of AT2R in RBF response to Ang II is gender related. In order to test this hypothesis, male and female rats were subjected to I/R injury and the RBF response to Ang II administration was measured while AT2R was blocked by PD123319.

## 2. Methods

### 2.1. Animals

The design of this experiment was approved by the Ethics Committee of the Isfahan University of Medical Sciences and followed the NIH guidelines for the treatment of animals. Male (209 ± 1.7 gr, *n* = 14) and female (185 ± 1.1 gr, *n* = 13) Wistar rats were housed in cages at a room with ambient temperature of 24 ± 1°C with light/dark cycle and fed with rat chow and water* ad libitum* for one week before running the experiment.

### 2.2. Surgical Preparation

The rats were anesthetized with urethane (1.7 g/kg i.p., Merck, Germany). The left jugular vein was isolated, ligated distally, and cannulated with polyethylene tubing (PE 9658, Microtube Extrusions, North Rocks NSW, Australia) for vehicle or antagonist administration. Left carotid and femoral arteries also were catheterized and the catheters were attached to a pressure transducer and a bridge amplifier (Scientific Concepts, Vic., Melbourne, Australia) for measuring mean arterial pressure (MAP) and renal perfusion pressure (RPP), respectively. The trachea was cannulated to facilitate ventilation. The rats were placed in a lateral position on a table with heating lamp to maintain body temperature at 37°  ±  0.5°C. The left kidney was exposed and placed in a cup secured to the operating table, and its artery was surrounded by ultrasound flow probe (Type 2SB, Transonic Systems, Ithaca, NY, USA) interfaced with a compatible flowmeter (T108, Transonic Systems) to measure RBF. An adjustable clamp was hooked around the aorta above the level of the renal arteries to induce renal ischemia and maintain RPP at control levels during infusion of Ang II. Throughout the experiment MAP, RPP, and RBF were measured continuously and data were taken as 2-second averages via a data acquisition system.

### 2.3. Experimental Protocol

#### 2.3.1. Antagonist Response

The animals were randomly divided into 2 groups of males (groups 1 and 2) and two groups of females (groups 3 and 4).

Following surgical preparation, a 45–60-min period was allowed for stabilization and control recordings of MAP, RPP, and RBF were commenced. To induce renal I/R, RPP was adjusted on ~25 mmHg for a period of 30 minutes via tightening the aortic clamp, and reperfusion was allowed by loosening it. After that the effects of either vehicle (saline, groups 1 and 3) or AT2R antagonist PD123319 (groups 2 and 4) were examined. With the beginning of reperfusion PD123319 (Sigma, St. Louis, MO, USA), dissolved in 0.9% w/v saline, was administered as a bolus dose of 1 mg/kg followed by continuous infusion at 1 mg/kg/h using a microsyringe pump (New Era Pump System Inc., Farmingdale, NY, USA). The antagonist or vehicle infused continuously throughout the study.

#### 2.3.2. Response to Ang II Infusion

Ang II was administrated 30 min after antagonist or vehicle started to infuse. Graded Ang II infusion (100, 300, and 1000 ng/kg/min) was commenced using a microsyringe pump. Each dose of Ang II was administered for 15 minutes. RPP was maintained at pre-Ang II infusion levels throughout the experiment by manipulation of the aortic clamp. When response of MAP reached equilibrium, we recorded that data as RBF and MAP responses to Ang II. MAP, RPP, and RBF responses were determined over the final 5 min of each infusion. The rats were sacrificed at the end of experiments via anesthetic overdose, and the left kidney was rapidly removed and weighed.

#### 2.3.3. Statistical Analysis

Data are expressed as mean ± SEM and were analyzed using the statistical software SPSS 20. Repeated measures' ANOVA was used to compare the effect of each treatment in male and female rats. RBF response to Ang II is also reported as percent change from the baseline values and was compared via repeated measures' ANOVA between the groups. *P* ≤ 0.05 was considered statistically significant.

## 3. Results

### 3.1. Baseline Measurements

The basal measurements as control for MAP, RPP, and RBF corrected for gr kidney weight (RBF/gKW) is shown in Table 1. No significant differences were detected between the vehicle and PD123319 treated groups in both genders.

**Figure 1:**
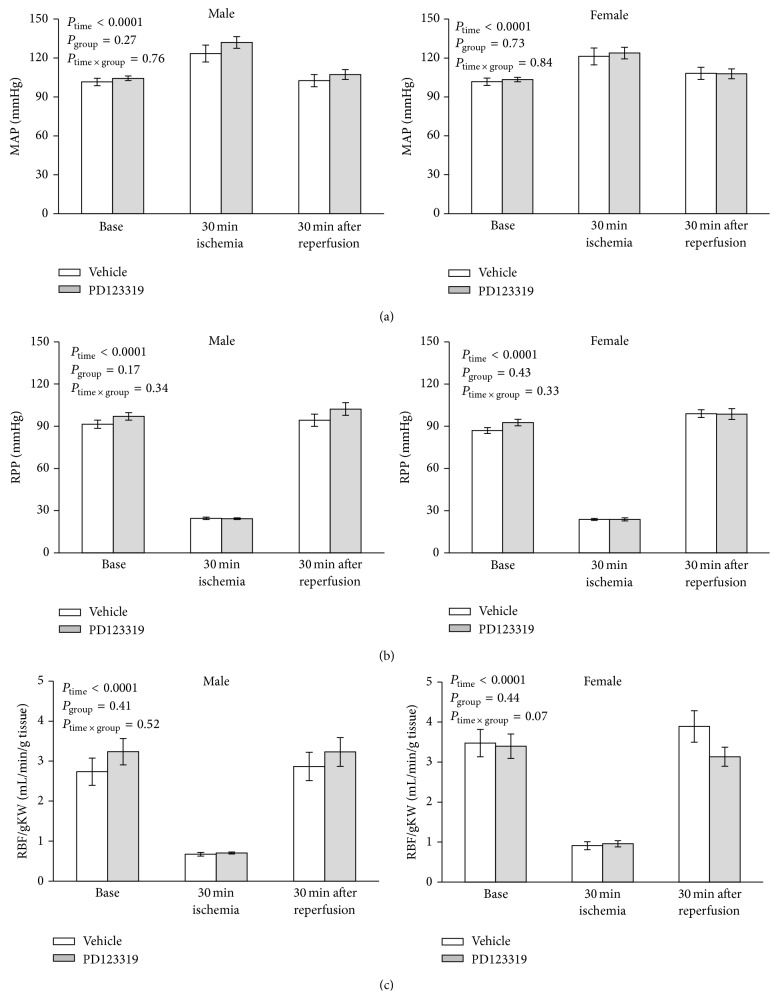
The hemodynamic parameters in male and female rats before ischemia (base), during renal ischemia, and 30 min after reperfusion in two groups of vehicle or AT2R antagonist, PD123319, treatment. The *P* values were derived from repeated measures ANOVA.

During the renal ischemia, MAP increased significantly; however no significant difference in MAP was observed between vehicle and PD123319 treated groups. As expected, RPP and RBF were decreased significantly during the renal ischemia, but no significant difference was detected between the groups (Figures 1(a)–1(c)).

Immediately after inducing reperfusion, the antagonist or vehicle started to infuse, and 30 min later (30 min post reperfusion) the effect of antagonist on MAP, RPP and RBF/gKW were determined. The results indicated that antagonist or vehicle administration has no significant effects on MAP, RPP, and RBF/gKW (Figures 1(a)–1(c)).

### 3.2. Responses to Ang II Infusion

**Figure 2:**
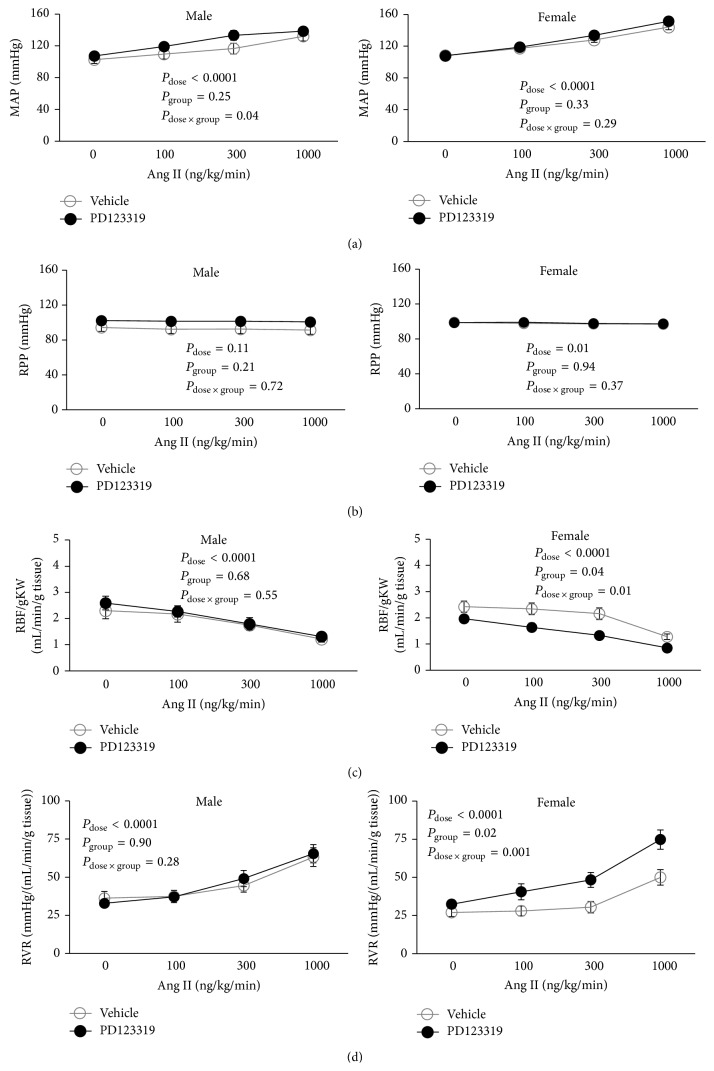
MAP, RPP, RBF, and RVR responses to Ang II administration which started to infuse 30 min after reperfusion. Data are shown as mean ± SEM. MAP, mean arterial pressure; RPP, renal perfusion pressure; RVR, renal vascular resistance; RBF, renal blood flow. *P* values were derived from repeated measures ANOVA.

**Figure 3:**
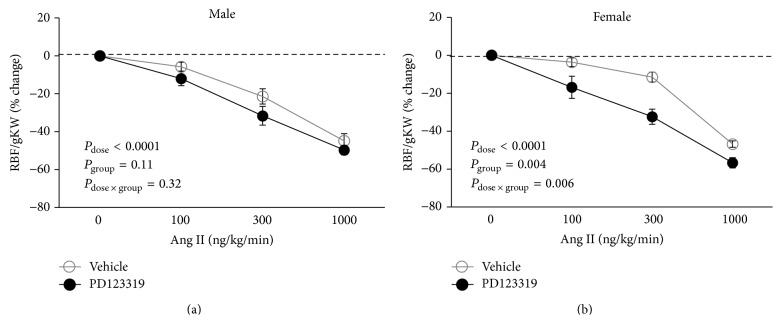
Percentage change of RBF responses to Ang II administration which started to infuse 30 min after reperfusion. Data are shown as mean ± SEM of percentage changes from the baseline (30 min after reperfusion assumed as a base). RBF, renal blood flow. *P* values were derived from repeated measures ANOVA.

Ang II infusion increased MAP in a similar dose-related manner in the vehicle or PD123319 treated male and female rats (Figure 2(a)). In all groups, RPP was kept relatively constant during Ang II infusion by the manipulation of the aortic clamp (Figure 2(b)). RBF/gKW was decreased in a dose-related manner in response to Ang II infusion (*P*_dose_ < 0.0001) in both genders; however in female rats PD123319 reduced RBF response to Ang II infusion significantly when compared with vehicle treated rats (*P*_group_ = 0.04, Figure 2(c)). Such observation was not seen in male rats, and PD123319 did not alter RBF/gKW response to graded Ang II infusion (Figure 2(c)). The percentage change of RBF/gKW response to Ang II infusion also is shown in Figures 3(a) and 3(b). More percentage change of RBF/gKW was observed in female rats treated with PD123319 when compared with vehicle treated animal (*P*_group_ = 0.004, Figure 3(b)). For example, in the females at 300 ng/kg/min of Ang II, RBF/gKW fell by 46 ± 1.6 and 56 ± 2.7 percent in the vehicle and PD123319 groups, respectively.

Finally calculated RVR (RPP/(RBF/gKW)) increased in a dose-related manner in response to Ang II infusion (*P*_dose_ < 0.0001). In the female rats, PD123319 significantly altered the RVR response to graded Ang II infusion when compared with vehicle treated group (*P*_group_ = 0.02, Figure 2(d)). However such observation was not seen in male (Figure 2(d)).

## 4. Discussion

The major finding of this study was in agreement with the hypothesis that RBF response to Ang II administration is gender related in I/R rat model.

It is documented that less than one-fifth of the Ang II binding sites can be related to the AT2R in the adult kidney [31, 32], and Ang II binds to the AT2R in the same affinity to the AT1R [33] while there is high affinity for PD123319 too [34]. AT2R is found in rat [35] and human [36, 37], and in humans, chromosome X contains AT2R gene [36]. This receptor exists in some organs such as brain [38], heart [39], kidney [40], and reproductive tissues [41]. The expression of AT2R was found in glomeruli and distal tubules of adult rats [42].

In rat kidney, the AT2R mRNA is distinguished from various tubular and vascular parts of the cortex and medulla, including the proximal tubule, collecting duct, arcuate arteries, afferent arterioles, and outer medullary descending vasa recta [40]. In humans, AT2R mRNA was found in vessels, tubular organization, and glomeruli [43]. Our results indicated that AT2R antagonist, PD123319, increased the RBF/gKW response to graded Ang II infusion in female after I/R. This result is in consistence with other studies that reported AT2R modulates renal function gender dependently [23, 44] which highlighted the vasorelaxant effect of AT2R on female. Indeed the vasorelaxant actions of the AT2R were attributed to NO production [45], and it is reported that this vasorelaxant action is related to activation of vascular kinin system while this receptor could even stimulate renal NO in bradykin B2-receptor-null mice [45–47]. I/R and its outcomes are sexually dimorphic in experimental disease models and sex steroid plays a predominantly role in the gender differences. Different studies demonstrated the effects of sex steroids on I/R injuries in animal models and firmly accepted the protective role of estrogen [10, 48–52] but the details of the mechanism are not well understood [53].

Also there is firmed evidence that AT2R probably is involved in the blood vasorelaxant action of AT1R antagonists especially in hypertensive experimental models [54, 55]. Accordingly, AT2R shows vasorelaxant action [56] and it is estrogen dependent because its expression was decreased in ovariectomized rats [57]. In addition, AT2R-induced relaxation requires XX chromosome sex complement addition to estrogen [58]. Similar to our result in I/R rat model, Hilliard et al. reported such observation on intact rat model and suggested this effect is partly attributable to the AT2R blockade ability of increasing AT1R-mediated effects of Ang II [20]. The effect of AT2R blockade also may be eminent in the preglomerular vasculature through which RBF and in turn the glomerular capillary pressure and glomerular filtration rate are reduced [24]. As we observed in present study RVR was increased in PD123319 treated female group. It is noteworthy that the greater renal tissue AT1R/AT2R ratio in male than in female rat [41] may provide more RVR in response to Ang II in the presence of PD123319. On the other hand, after ischemia, the new expression of renal AT2R mRNA in outer medulla and proximal tubules [30] may be blocked by PD123319 to promote the vasoconstriction response to Ang II administration. The gender difference obtained in RBF response to Ang II may relate to some other factors. Female sex hormones increase endothelial NO synthase (eNOS) in renal medullary [14, 23], and eNOS attributes to AT2R in the kidney [59] that mediates vasodilatory effects in the kidney by NO production that depended on bradykinin [60] and PGE2 formation [61]. The AT2R vasodilator effects also may obtain by endothelium-derived hyperpolarizing factors (EDHFs) which are predominate in women [58] and this factor increases in I/R too [62]. Finally the other receptor from RAS, Mas receptor, may involve I/R injury. It is reported that expression of the Mas receptor increases at 4 h of reperfusion after I/R injury [63]; however the exact mechanism needs to be defined.

## 5. Conclusion

During kidney ischemia, the reduction of RBF disturbs renal function, and, in this study, the important role of AT2R in renal circulation was evident in female rat induced kidney I/R injury. The stimulation of this receptor may be considered for the treatment of RBF in condition of I/R injury.

## Figures and Tables

**Table 1:** Baseline data for MAP, RPP, RBF, and RBF corrected for gr kidney weight.

Group	Male				Female			
	MAP	RPP	RBF	RBF/g KW	MAP	RPP	RBF	RBF/g KW
Vehicle	101.4 ± 2.8	91.4 ± 2.9	2.18 ± 0.30	2.73 ± 0.34	101.7 ± 1.2	86.9 ± 2.1	2.17 ± 0.21	3.47 ± 0.34
PD123319	104.3 ± 1.7	97.0 ± 2.6	2.60 ± 0.24	3.24 ± 0.33	103.4 ± 2.4	92.6 ± 2.3	2.13 ± 0.18	3.39 ± 0.30
*P*_*t* test_	0.45	0.19	0.34	0.33	0.53	0.10	0.87	0.89

MAP: mean arterial pressure, RPP: renal perfusion pressure, RBF: renal blood flow, and RBF/g KW: renal blood flow *per* gram of left kidney weight.
